# The Roles of Semiochemical Perception and Surface Compounds in a Pharmacophagous Sawfly

**DOI:** 10.1007/s10886-025-01676-1

**Published:** 2025-12-15

**Authors:** Leon Brueggemann, Gina S. Fleer, Caroline Müller

**Affiliations:** 1 Department of Chemical Ecology, Bielefeld University, Universitätsstr. 25, Bielefeld, 33615 Germany; 2 Joint Institute for Individualisation in a Changing Environment (JICE), University of Münster and Bielefeld University, Bielefeld, Germany

**Keywords:** Cuticular hydrocarbons, Chemical communication, Hymenoptera, Perception, Pharmacophagy, Surface compounds

## Abstract

Pharmacophagy is a fascinating phenomenon, where animals take up specialized plant metabolites unrelated to nutrition, but with benefits for, for example, defense and mating. Adults of the turnip sawfly *Athalia rosae* engage in pharmacophagy of *neo*-clerodane diterpenoids (clerodanoids) from the bugleweed *Ajuga reptans*, but can also take up metabolites sequestered from the plant and modified from conspecifics. Here, we investigated the perception of odors associated with pharmacophagy from long and close distances in bioassays, using leaves and washes of surface compounds, mainly cuticular hydrocarbons, from female and male adults of different pharmacophagy treatments. Extracts were analyzed by GC-MS to test whether the treatment affected the cuticular surface composition. We hypothesized that sawflies perceive compounds related to pharmacophagy at close distances, that sexes differ in surface compound profiles, and that contact with *A. reptans* leaves or previously *A. reptans*-exposed conspecifics alters the cuticular hydrocarbon composition. Our results demonstrate that sawflies indeed perceive both plant leaves and surface washes from previously *A. reptans*-exposed conspecifics at close distance. We characterized the major cuticular hydrocarbons of *A. rosae* and uncovered sexual dimorphism in surface compound abundance, but found no pharmacophagy-induced changes in the surface compound composition. We discuss that other compounds than cuticular hydrocarbons are likely involved in the observed effects. Our research provides insights into how specialized plant metabolites may drive the evolution of the insect sensory behavior and into the complexity of insect chemical communication.

**Supplementary Information:**

The online version contains supplementary material available at 10.1007/s10886-025-01676-1.

## Introduction

Communication between organisms is ubiquitous and occurs through various channels, with chemical signalling being considered one of the most ancient forms (Mithöfer and Boland 2016). Chemical communication serves as a multifaceted channel for mediating interactions among organisms and with their environments, whereby chemical cues are often combined with, e.g., visual cues to increase the reliability and specificity (Guignard et al. 2022; Rypstra et al. 2009). A major role in chemical communication is played by semiochemicals, which regulate not only intraspecific behaviors such as mating or aggregation, but also interspecific relationships across trophic levels, shaping interactions among plants, herbivores, predators, and microbes (Mbaluto et al. 2020). Infochemicals, including semiochemicals, are recognized as ecological drivers that show effects on ecosystem scales and can mediate processes such as competition, mutualism, and habitat selection (Zu et al. 2023). Furthermore, they function as crucial mediators of niche realization processes by modulating behavioral and physiological traits in both social and solitary species (Müller et al. 2020). The role of specific semiochemicals has been studied in various species and species interactions, but little is known about chemical communication in species that engage in pharmacophagy, where animals take up and sequester plant compounds for purposes other than nutrition (Boppré, 1984); Singh and Müller 2025).

Chemical cues in insects are often encoded on the cuticle in surface compounds, particularly cuticular hydrocarbons (CHCs), which serve multiple roles in physiological and ecological contexts. First mainly described as desiccation barriers that prevent water loss through the insect cuticle, CHCs have since been widely recognized as semiochemicals critical for intra- and interspecific communication (Blomquist and Bagnères 2010). CHCs are straight-chain, methyl-branched, either saturated or unsaturated hydrocarbons, which can convey important information related to, for example, species, sex, and colony (Howard and Blomquist 2005). In various social but also solitary insect species, CHCs are involved in conspecific recognition or mate choice, with specific CHC blends acting as contact or sex pheromones (Moris et al. 2025; Nascimento et al. 2013; Tyler et al. 2015). CHC profiles can be influenced by internal factors such as genetics, physiology, and age, as well as by external factors such as temperature, humidity, and diet (Chung and Carroll 2015; Otte et al. 2018). Notably, changes in CHC profiles can reflect interactions with specific food sources or specialized plant metabolites (Geiselhardt et al. 2012; Piskorski et al. 2010; van Wilgenburg et al. 2022). However, whether pharmacophagy can modulate the perception of surface compounds and modulate their composition is not fully understood.

Utilizing the encompassed information of such semiochemicals requires prior detection. The volatility of semiochemicals and their effect on the receiver can differ even within species (Birkett et al. 2004; Ehlers et al. 2021). Volatile compounds such as, for example, green leaf volatiles or terpenoids from host plants, can be already perceived from the distance (Boncan et al. 2020; Metcalf 1987; Pichersky and Raguso 2018). The response to such compounds by herbivores is often tested in olfactometer assays (Roberts et al. 2023). In contrast, compounds with lower volatility such as CHCs may be perceived by taste sensilla located at labial palps and other mouthparts on close distance, as described in *Drosophila melanogaster* (Lacaille et al. 2007; Ozaki et al. 2010). To test these reactions, close distance observations of behavior are needed.

*Athalia rosae* (Hymenoptera: Tenthredinidae) is an holometabolous insect species that interacts with different plant families. While larvae feed on Brassicaceae, adult *A. rosae* take up nectar of e.g. Apiaceae (Bandeili and Müller 2010; Saringer 1976). Beyond their nutritional plant interaction, the adults engage in pharmacophagy, where they acquire *neo*-clerodane diterpenoids (clerodanoids), such as areptin A and areptin B, from non-food plants such as *Ajuga reptans* (Lamiaceae) (Brueggemann et al. 2023). The clerodanoids have been described to be stored in glandular trichomes on the leaf surface in other species (Amano et al. 1999; Wang et al. 2021). When taken up by *A. rosae*, the clerodanoids are subsequently hydrolized (Brueggemann et al. 2023) and then utilized to gain benefits in mating, and to deter predators (Amano et al. 1999; Paul and Müller 2022; Singh et al. 2022). In addition, these compounds mediate interactions within *A. rosae* through kleptopharmacophagy, whereby individuals take up these compounds from previously *A. reptans*-exposed conspecifics through licking (previously also called nibbling) on the surface (Singh et al. 2024). The clerodanoids were found on the surface of *A. rosae*, mediating this behavior in conspecifics (Brueggemann et al. 2023).

The present study aimed to investigate the long- and close-distance responses of semiochemical perception related to the phenomenon of (klepto-)pharmacophagy in *A. rosae*. We tested the long distance responsiveness of adults towards *A. reptans* leaves in a Y-olfactometer assay and the perception to pharmacophagy-related cues in a close distance assay, using a novel set-up (modified from Arican et al. 2020 and Kuwabara et al. 2023) for testing maxilla-labia responses to different stimuli. Sawflies were either unexposed, directly exposed to an *A. reptans* leaf or had contact to a previously exposed conspecific. Surfaces washes of adults with these different pharmacophagy treatments were analyzed via gas chromatography coupled with mass spectrometry. We expected sawflies to respond to *A. reptans* leaves and surface washes of exposed conspecifics on closer distances in a range of around 5 cm. Regarding surface compound composition, we hypothesized that females and males would differ in their surface compounds but also due to the engagement in pharmacophagy, which may change the chemical phenotype of the sawfly.

## Materials and Methods

### Insect and Plant Rearing

Adult individuals of *A. rosae* were collected over summer and early autumn in the surroundings of Bielefeld University (Germany) and introduced in our laboratory culture. Adults were offered *Sinapis alba* (Brassicaceae) plants for oviposition and larvae were provided with non-flowering plants of *Brassica rapa* var. *pekinensis* (Brassicaceae) as host plants. Insects were kept in cages at a 16:8 h day: night cycle. Larvae were provided with soil for pupation. Emerging adult individuals were separated by sex, provided with 2% honey water and kept at ~ 5˚C in the dark until they were used to mate and offered plants for oviposition. Plants of *A. reptans* were collected around the university and kept in a greenhouse with a 16:8 h day: night cycle without a specific climate control.

### Testing Perception of Volatiles from a Long Distance

To test for volatile perception by adult *A. rosae* from a long distance, a Y-tube olfactometer was used. The Y-tube was made from glass and had a base tube length of 8 cm, with the Y-arms of 7 cm length and a diameter of 10 mm. The Y-tube was connected by Teflon tubing to two glass cylinders (length 10 cm; diameter 25 mm) in which the odor sources were offered. The airflow was maintained with a 3 V air pump and regulated at 0.1 LPM to ensure that plant volatiles could reach the individual without hindering its movement. Incoming air was filtered through water with activated charcoal to eliminate external odors. The Y-tube was positioned at an angle of 45° and a lamp centrally illuminated the top of the Y-tube. Side panels around the set-up were used to exclude external stimuli. Unmated, adult sawflies were placed individually at the base of the Y-tube and observed for five minutes. Once they entered one Y-arm for at least 0.5 cm, this was scored as a decision for that volatile source. To test that no side-preferences exist, first assays were done without offering any stimuli. Here, a comparable number of individuals chose for either arm. To test the response to odors of the host plant (cabbage) and to *A. reptans* odors, leaf parts (cabbage) or two middle aged leaves (*A. reptans*) were placed in one glass tube and the other was left empty. The Y-tube was thoroughly cleaned with acetone and heated to 200 °C between trials to remove residual odors and stimuli were switched every other test run. Overall, 30 females and 30 males were tested per plant species and each individual was only tested once.

### Testing Perception of Compounds at a Close Distance

**Fig. 1:**
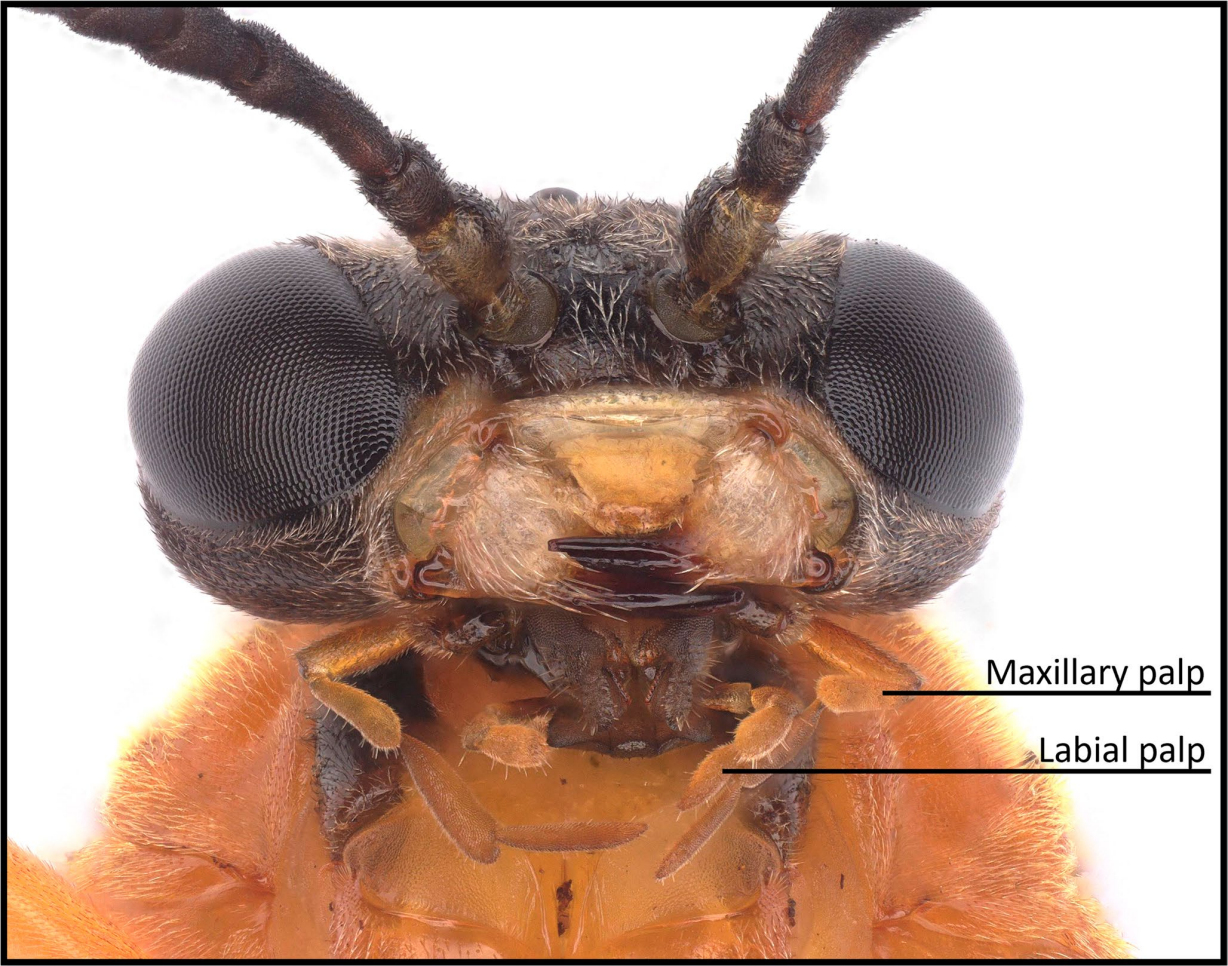
Close-up of head with mouthparts of *Athalia rosae*. Movements of the maxillary and labial palps were observed in the maxilla-labia response assay. Picture taken by Marco Niekampf

To test for compound perception by *A. rosae* at a close distance, one day old adults (*n* = 12 females and *n* = 12 males) were placed individually in Petri dishes (5.5 cm diameter), in which four different stimuli were offered subsequently in a randomized order, with a 10 min break in between. The stimuli were either an empty glass slide (15 × 15 mm), a slide with 10 µl of a surface wash from a C- individual (equivalent to 1/10 of a wash from one animal; for preparation of washes see **Preparation of Surface Washes and Analyses**), a slide with 10 µl of a surface wash from a C + individual, or a piece of a *A. reptans* leaf (about the same size as the glass slide). The surface extracts were taken from different individuals and the sex of the extracted individual was matched to the test individual. The stimuli were always placed in the center of the Petri dish. The total time spent licking on the slide/leaf was recorded for 3 min. Two hours later, a maxilla-labia response assay, modified from Arican et al. (2020) and Kuwabara et al. (2023), was performed. Therefore, reaction tubes (1 ml) were cut in half at the tip and one side removed. A sawfly was introduced in the tube with the head positioned in the open tip of the tube. Parafilm was used to fix the body but allow the head to move freely. The fixation of the sawflies was done at 4 °C. After fixation, each sawfly was placed at room temperature (20 °C) for 20 min to acclimatize. Following the acclimatization, the same stimuli as in the Petri dish assay were presented in a 3 mm distance to the sawfly mouthparts in a randomized order to score for a direct reaction, i.e., rigorous movement, of the maxillary and the labial palps (Fig. 1). Each stimulus was offered for 10 s, with a 5 min break in between. Each individual was offered all four stimuli first in the Petri dish and then in the maxillary-labia response assay.

Since the surface washes contained the internal standard *n*-eicosane, an additional test series was performed to investigate the response to this compound. The solvent control (dichloromethane), *n*-eicosane in dichloromethane (0.01 mg/ml), and C + surface extracts as positive controls were tested in the same way as described above in both the Petri dish assay and the maxillary-labia response assay (*n* = 5 females and *n* = 5 males).

### Preparation of Surface Washes and Analyses

**Fig. 2:**
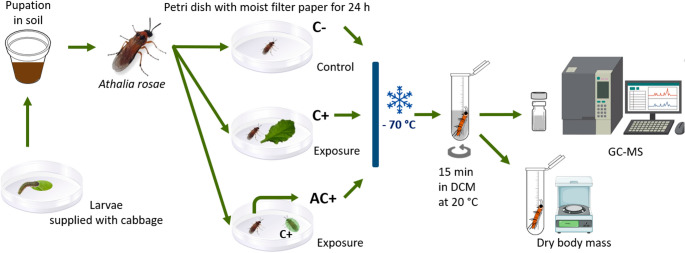
Experimental setup and exposure to treatments for the surface compound analysis. DCM – dichloromethane

A mixed group of 30 mated and unmated adult female sawflies was placed in a cage for oviposition. The offspring was provided with cabbage *ad libitum*. After molting into their last instar, the larvae were separated into individual pots with soil for pupation. After adult emergence, adults that had hatched within the last 20 h were taken and placed individually into Petri dishes (5.5 cm diameter) lined with moist filter paper. For different pharmacophagy treatments, one subset of sawflies was kept there for 24 h without exposure to *A. reptans* leaves and thus no clerodanoid access (C-; *n* = 8 females and *n* = 17 males). Another subset of the individuals received access to a disc (6 mm diameter) of a fresh middle-aged leaf of *A. reptans* for 24 h, providing thus direct exposure to plant clerodanoids (C+; *n* = 8 females and *n* = 17 males). A third subset of individuals was provided contact for 24 h to another sawfly of the same sex that had been in contact with a leaf for 24 h, providing thus exposure to clerodanoids incorporated by conspecifics (AC+; *n* = 8 females and *n* = 13 males; Fig. 2). No food was provided in this entire period. Each treatment ended after 24 h with freezing the sawfly in a 1.5 ml reaction tube at −70 °C, leading to a quick death and preservation of surface compounds.

Samples were thawed at room temperature for 15 min. Then, 100 µl of dichloromethane (Merck, Germany) with *n-*eicosane (0.01 mg/ml, Thermo Fisher, USA) as internal standard were added to each sample and samples mixed for 15 min at room temperature. Dichloromethane was chosen to cover a broader range of surface compounds and also include more polar compounds (as also performed by Geiselhardt et al. 2009 and Ruther et al. 2011). These extracts were pipetted into vials with glass insert and stored again at −70 °C. Afterwards, the sawflies were dried at 50 °C overnight and weighed to determine their dry body mass.

To analyze the surface compounds, the extracts were measured using a GC-MS (QP2020, Shimadzu, Japan) with a VF-5 MS column (30 m × 0.2 mm inner diameter with a 10 m guard column, Varian, USA). Electron impact ionization mode was set at 70 eV and helium was used as carrier gas with a flow rate of 1.5 mL min^− 1^. The initial temperature of 100 °C was increased to 150 °C at 10 °C per min, further to 325 °C at 8 °C per min, finally held for 5 min. Solvent blanks including the internal standard as controls and an alkane standard mix (C7–C40, Sigma Aldrich, Germany) were analyzed using the same method. Compounds were putatively identified by comparing the linear retention indices (LRI, van den Dool 1963), calculated based on the alkane series, and mass spectra with those of available reference data from the National Institute of Standards and Technology (NIST Database 14 and webpage, 2025). Further spectral data and LRI of Hymenoptera surface compounds were used as additional references (Bartelt et al. 2002; Soares et al. 2017). For linear alkanes (C8 –C40), standards could be used for identification and verification. Surface compound data were initially processed using GCMS Postrun Analysis (Shimadzu, Japan). For quantification, peak areas based on total ion chromatograms were divided by the peak area of the internal standard and sample dry mass, followed by blank subtraction.

After GC-MS analyses, the extracts were frozen again at −70 °C and then thawed for 15 min before use in the bioassays (see **Testing Perception of Compounds at a Close Distance**).

### Statistical Analysis

All statistical analyses were performed in RStudio version 4.4.2 (R Development Core Team 2024). For the Y-tube olfactometer assay a binomial test was used. The total licking time at the different stimuli in the Petri dish assay was compared with a Friedman test, followed by pairwise Wilcoxon signed-rank tests for paired data with Bonferroni correction. The number of individuals showing a response with their palps in the maxilla-labia response test was analyzed with a Fisher’s exact test.

To visualize differences in surface compound profiles, non-metric multidimensional scaling (NMDS) was performed using the metaMDS function and the beta dispersion was calculated (vegan package version 2.6–10) based on Bray–Curtis dissimilarities (Oksanen et al. 2025). Differences in surface compound composition and beta dispersion between treatment groups were tested using permutational multivariate analysis of variance (PERMANOVA) via the adonis function from the same package (Oksanen et al. 2025) and Tukey-HSD. Random forest analysis (number of trees = 500) was used to uncover compounds that led to the strongest separation. Peak areas (normalized to internal standard and body mass) of the three compounds that explained most of the clustering between sexes in the NMDS were tested for differences with Mann Whitney-U tests. Data visualization was performed using the ggplot2 package (version 3.5.1 Wickham 2016).

## Results

### No Attraction to *Ajuga reptans* Odor in Y-Tube Olfactometer

**Fig. 3:**
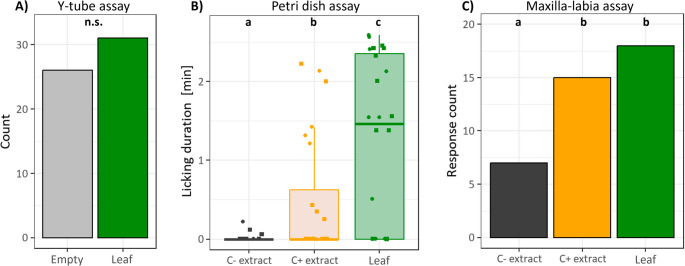
Reaction of adult *Athalia rosae* to stimuli at greater distance in Y-tube olfactometer assay (**A**), at closer distance in the Petri dish assay (**B**) and in the maxilla-labia response assay (**C**). (**A**) Number of sawflies (*n* = 57, females and males together, NA excluded in figure) choosing either the empty side or the side with the *Ajuga reptans* leaves (binomial test). (**B**) Licking duration in petri dish assay for female (dots) and male (square) adults (*n* = 24 in total). Boxplots show interquartile ranges (IQR, boxes) with medians, whiskers extent to ± IQR*1.5, and raw data points. Data was tested with a Friedman test, followed by pairwise Wilcoxon signed-rank tests with Bonferroni correction. None of the individuals licked on the clean glass slides, therefore this data is not presented. (**C**) Number of individuals showing maxilla-labia responses to different stimuli (Fisher’s exact tests). Different letters denote significantly different (*p* ≤ 0.05) reaction to stimuli

Female sawflies choose the arm with the cabbage odor significantly more often (binomial test, *p* = 0.043, *n* = 30), while males did not show a preference (*p* = 0.362, *n* = 30) (Fig. S1). However, adults of both sexes did not show an olfactory preference for *A. reptans* leaves over clean air in the Y-tube olfactometer (binomial test, *p* = 0.597, *n* = 30 per sex; Fig. 3A). Of the tested individuals, 95% decided for either arm (27 females, 30 males; three females did not make a decision).

### Reaction to Conspecific and Plant Stimuli at Close Distances

In the Petri dish assay, adults showed significant differences in licking time on the different stimuli (Friedman test *ꭓ*^*2*^ = 16.82; *df* = 2; *p* < 0.001). Adults did not lick at all on clean glass slides and only three showed short licking on C- extracts. They licked on C + extracts for longer time periods than on C- extracts (pairwise Wilcoxon signed-rank test, *V* = 6, *p* = 0.032; Fig. 3B). Adults licked longest on the *A. reptans* leaf (pairwise Wilcoxon signed-rank test with other two stimuli, *V* = 19, *p* < 0.021). The data for females and males were similar and therefore pooled to increase sample size for testing.

In the maxilla-labia response assay, adults also showed significantly different responsiveness to the different stimuli (*df* = 2, *p* = 0.005, Fig. 3C). No extension of the palps was shown towards control slides without a stimulus. Few sawflies reacted to C- surface washes, while more than twice as many reacted to C + washes and leaves.

Towards *n*-eicosane alone, sawflies did not show any response, reassuring that there were no side effects of the internal standard added to the surface washes in the performed trials (Fig. S2).

### Surface Compounds Differ between Sexes but not between Individuals of Different Pharmacophagy Treatments

In the surface washes, 35 distinct peaks were found (Fig. S3), of which the 15 most abundant compounds could be putatively identified (Table 1). The majority of these compounds are CHCs, namely long-chain alkanes, alkenes, or methyl-branched alkanes. 1-Docosanol can be classified as a fatty alcohol because of its hydroxy group.

**Table 1:** Most abundant surface compounds of *Athalia rosae.* Compounds were putatively identified via the NIST database and linear retention indices (LRI)

Putative ID	RT	Sum formula	Molecular mass	LRI
Tricosane	16.78	C_23_H_48_	324	2300
11-Methyltricosane^1^	17.14	C_24_H_50_	338	2335
Tetracosane	17.82	C_24_H_50_	338	2400
7-Pentacosene	18.16	C_25_H_50_	351	2434
9-Pentacosene	18.45	C_25_H_50_	351	2463
1-Docosanol	18.54	C_22_H_46_O	326	2472
Pentacosane	18.83	C_25_H_52_	352	2500
11-Methylpentacosane^1^	19.15	C_26_H_54_	367	2534
5-Methylpentacosane^1^	19.30	C_26_H_54_	367	2549
3-Methylpentacosane^1^	19.53	C_26_H_54_	367	2573
3-Methylhexacosane^1^	20.08	C_27_H_56_	381	2631
4,8-Dimethylhexacosane^1^	20.56	C_28_H_58_	395	2682
Heptacosane	20.73	C_27_H_56_	380	2700
11-Methylheptacosane^1^	21.00	C_28_H_58_	395	2731
4-Methyltritriacontane^1^	26.15	C_34_H_70_	479	3358

^1^ Methyl group positions are putative

**Fig. 4:**
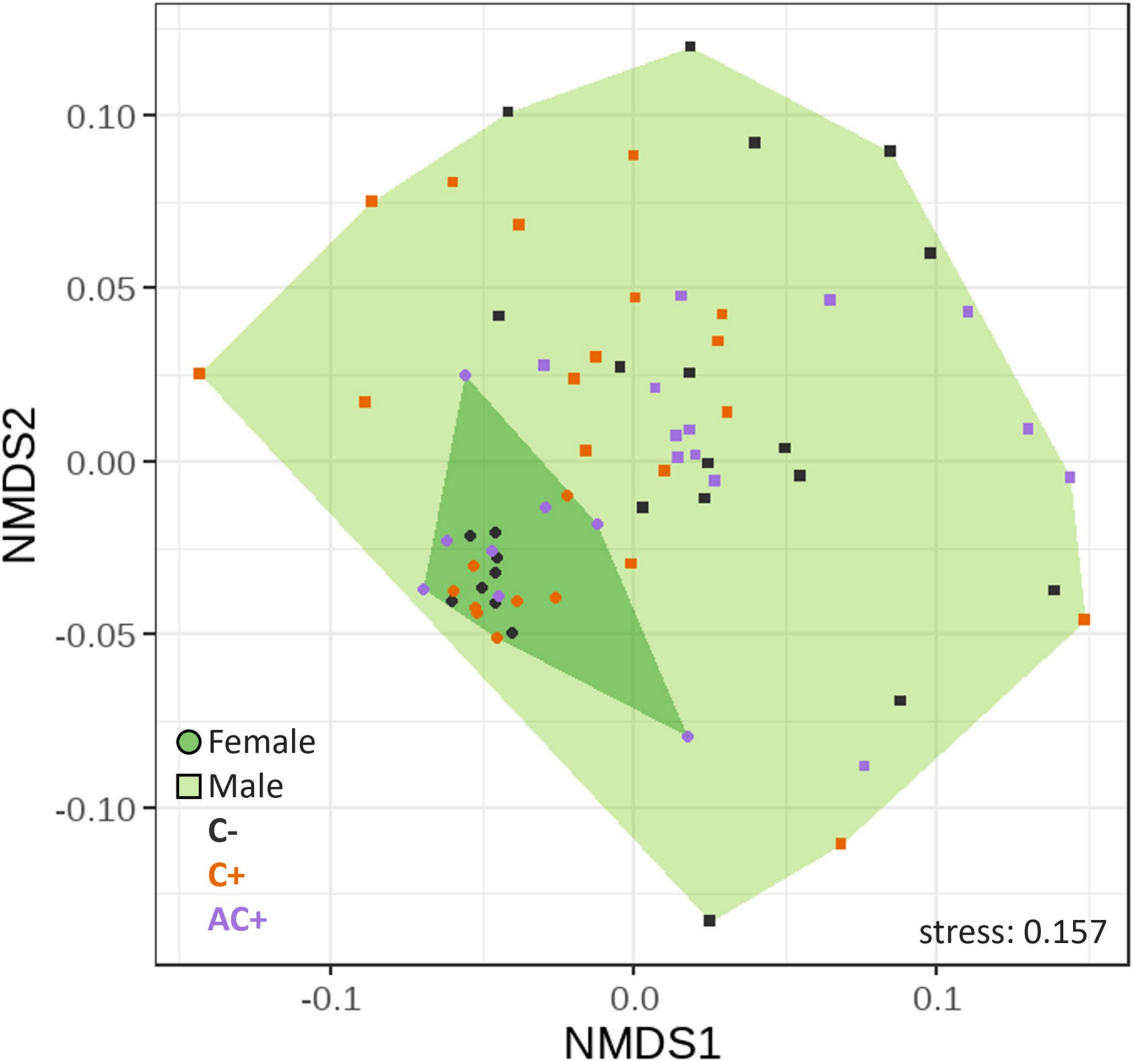
Non-metric multidimensional scaling (NMDS) of 35 compounds found in surface washes of *Athalia rosae* adults, measured by GC-MS, based on Bray-Curtis distances. Males and females exposed to different pharmacophagy treatments were measured: C- no contact to clerodanoids, C + clerodanoids through *Ajuga reptans* leaves, AC + contact to clerodanoids through another exposed conspecific. Convex hulls indicate differences between male (light green) and female (dark green) surface samples

The composition of surface compounds differed between sexes (*F* = 17.999, *df* = 1, *p* = 0.001) but neither due to pharmacophagy treatment (*F* = 1.325, *df* = 2, *p* = 0.194) nor due to the interaction of sex and treatment (*F* = 0.940, *df* = 2, *p* = 0.506; Fig. 4). Data of male washes showed a higher variability than those of females, the latter clustering closer together.

**Fig. 5:**
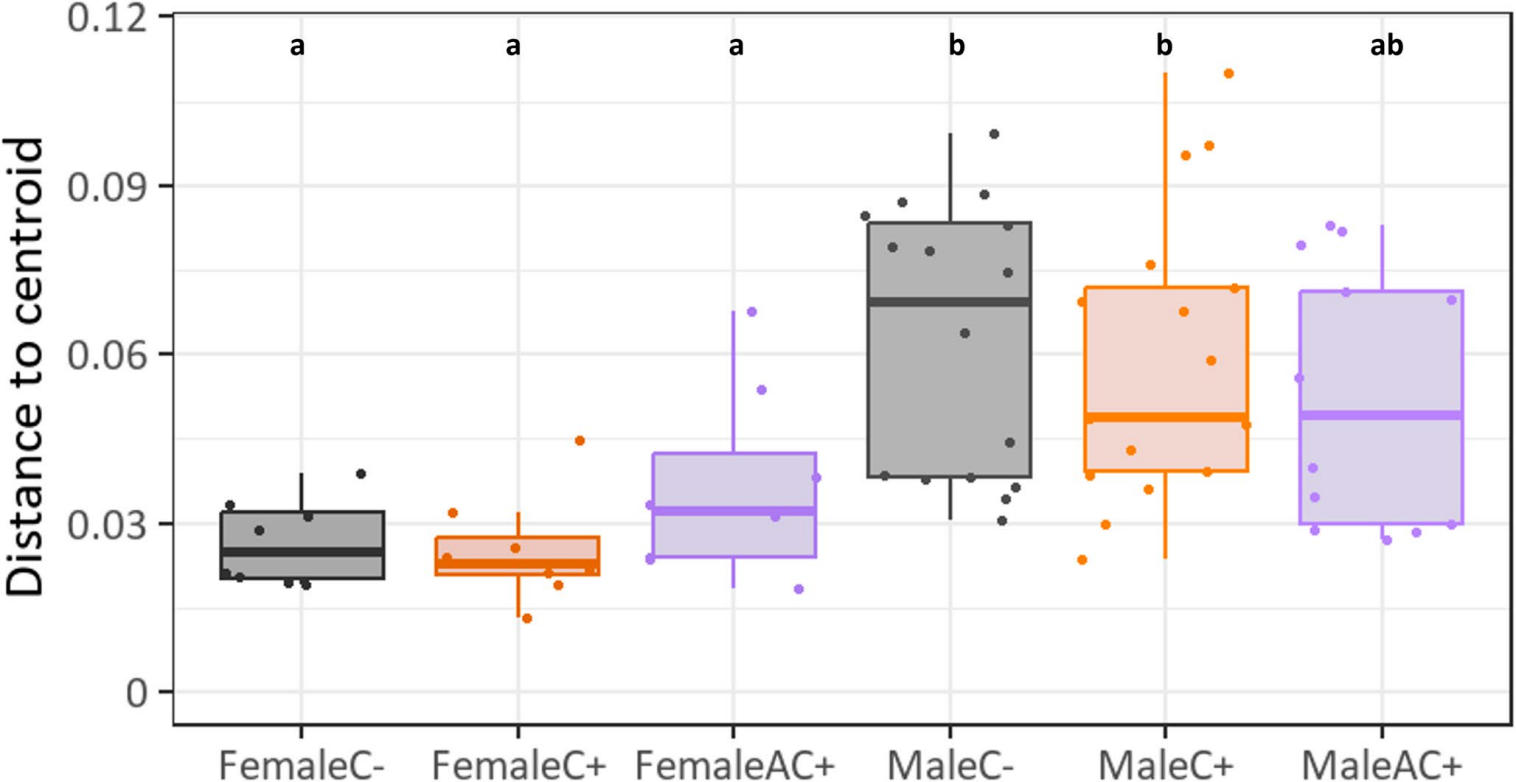
Beta dispersion of composition of compounds found in surface washes of adult *Athalia rosae* females and males of different treatments: C- no contact to clerodanoids, C + clerodanoids through *Ajuga reptans* leaves, AC + contact to clerodanoids through another exposed conspecific. Boxplots show interquartile range (IQR, boxes) with medians, whiskers extent to ± IQR*1.5, and raw data points (dots) of the Bray-Curtis distances to the centroids. Overall significant differences were first tested with ANOVA followed by Tukey-HSD. Different letters denote significantly different (*p* ≤ 0.05) beta dispersion

Female surface compositions generally showed lower distances to group centroids, indicating more consistent compositions and lower dispersion compared to male surface compositions (Fig. 5). Overall, the beta dispersion was significantly lower for females than males, except for the surface washes of the males of the AC + treatment.

**Fig. 6:**
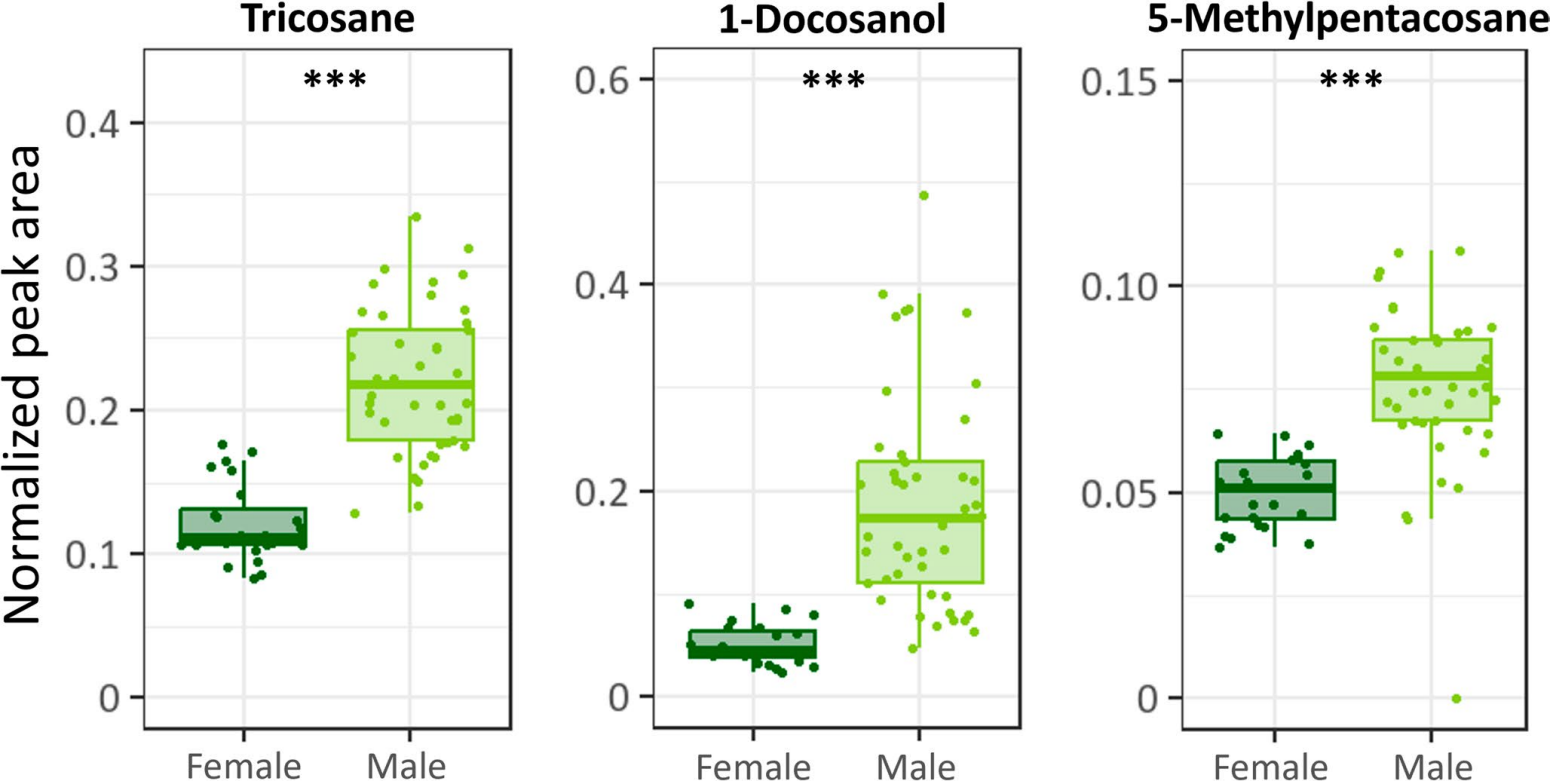
Normalized peak areas of compounds of surfaces washes of *Athalia rosae* adults differing most between sexes. Boxplots show interquartile range (IQR, boxes) with medians, whiskers extent to ± IQR*1.5, and raw data points (dots). Significant differences were tested with Mann-Whitney U tests (***: *p* ≤ 0.001)

Random forest analysis revealed three compounds that contributed most to the separation between sexes (Fig. S4). These compounds were putatively identified as tricosane, 1-docosanol, and 5-methylpentacosane. All three compounds were less abundant in females than in males (Mann Whitney-U test, W = 33, *p* < 0.001; Fig. 6).

## Discussion

Previous studies provided insights into the process of pharmacophagy, described the presence of relevant clerodanoids in *A. reptans*, and documented their uptake by adult turnip sawflies (Brueggemann et al. 2023; Singh et al. 2024). In field and laboratory studies we observed that adult sawflies exhibit rapid and selective responses to both *A. reptans* plants and conspecifics previously exposed to clerodanoid-containing tissues, suggesting the chemosensory perception over close distances and the recognition of conspecifics. To elucidate these processes, we here used a combination of behavioral bioassays and chemical analyses to test if compounds are perceived at a distance by adult sawflies and if pharmacophagy alters CHC composition.

Our results from the Y-tube olfactometer assay suggest that volatiles released from *A. reptans* leaves could not be recognized, or at least did not evoke a positive reaction, at a distance greater than 5 cm. Many herbivorous insects use specific blends of plant volatiles to find their preferred plants, probably because specific blends allow for greater flexibility in their odor coding systems (Bruce and Pickett 2011). Plants of *A. reptans* are known to emit a blend of volatiles dominated by 1-octen-3-ol (Frezza et al. 2019), which acts as an attractant for different insect species, but as a repellent for others (Xu et al. 2015). In contrast, the clerodanoids occurring in *A. reptans* may not be very volatile due to their structure and thus may only be sensed at close distance (Camps and Coll 1993; Coll and Tandrón 2007). Since females were attracted in the Y-tube olfactometer by the odors of the plant used for egg laying, cabbage, the functionality of our set-up was confirmed.

The Petri dish assay and the maxilla-labia response assay revealed that, while sawflies reacted to all tested stimuli except the control, they showed significantly more responses to washings from C + adults than C- washings. They also licked longest on *A. reptans* leaves, indicating a close-distance perception that may directly stimulate the maxillary palps and eventually lead to the uptake of compounds. The longer licking duration on leaves compared to washings of C + adults may be due to the higher concentration of clerodanoids in the leaves (Brueggemann et al. 2023) and potentially also to a higher diversity in clerodanoids present in *A. reptans* (Malakov and Papanov 1998). However, already one clerodanoid, clerodendrin B, has been shown to be sufficient to evoke feeding stimulation in *Athalia* (Nishida et al. 2004; Opitz et al. 2012) and may cause addictive behavior (Wink 2018).

In nature, we expect a combination of visual and olfactory stimuli to help locate sources of specialized plant metabolites (Guignard et al. 2022). Our Y-tube olfactometer setup excluded the visual channel to specifically test olfactory senses. In the Petri dish and the maxilla-labia response assay the green leaves also provided a visual cue, probably enhancing the stimulation of the adults. Nevertheless, also the colorless C + extracts on the slides evoked a licking response, demonstrating that chemical cues are sufficient here. The detection of relevant compounds probably occurs at olfactory antennal sensilla, as previously described in bumblebees (Mertes et al. 2021; Palottini et al. 2025). Following the perception of the compounds, *A. rosae* exhibits a licking behavior, sometimes referred to as nibbling (Brueggemann et al. 2023; Paul and Müller 2022). Our maxilla-labia response setup facilitated detailed observations of the mouthparts. The maxilla-labia palps made gentle contact with the leaf surface or extract on glass slides without causing visible damage. Based on these observations, we propose that “licking” more accurately describes this behavior than “nibbling,” as the interaction involves surface contact for compound acquisition, but no tissue damage. Additionally, we observed that the licking is sometimes followed by a cleaning or “body-lotioning” behavior, which may indicate a combination of internal metabolism and distribution on the surface.

Our analysis of *A. rosae* surface compounds revealed a CHC profile dominated by straight-chain alkanes, methyl-branched alkanes, alkenes, and one alcohol. The occurrence of these four compound groups is consistent with patterns documented across other Hymenoptera, such as eusocial wasps (Soares et al. 2017), and more specifically other Symphyta, such as the wheat stem fly *Cephus cinctus* (Bartelt et al. 2002). Next to these highly abundant compounds, we found 20 other compounds that were present only in low amounts and therefore could not be conclusively identified. The CHC profiles of many Hymenopteran species show considerable diversity, yet common structural patterns emerge within major taxonomic groups (Kather and Martin 2015). However, by using dichloromethane for the surface washes we may not have extracted some of the non-polar CHCs. Future analyses may thus be performed with other solvents such as hexane, as often used for CHC extraction (Howard and Blomquist 2005). Extracts may also be analyzed more concentrated to detect potential further compounds that are only present in minute concentrations. Our analyses revealed semi-quantitative, but not qualitative, differences in CHCs between females and males. Such a pattern is well-documented in other Hymenoptera species (Buellesbach et al. 2018). The differences in cuticular composition were mainly driven by three compounds that were, normalized to body mass, more abundant in males. In adults of *C. cinctus*, a pronounced sexual dimorphism exists, with (*Z*)−9-tricosene accounting for approximately half of the total CHCs in males but being nearly absent in female CHCs (Bartelt et al. 2002). The higher dispersion in surface compound composition of male *A. rosae* compared to females may reflect the selective pressures of male-male competition for mating opportunities, as CHCs are known to play an important role in sexual selection and mate recognition in various insect species (Lane et al. 2016; Mitchell et al. 2023; Moris et al. 2025). The lack of a significant difference in beta dispersion between AC + females and AC + males might have implications for mating preferences. Similar variability in females and males could balance the range of “acceptable” surface profiles, potentially reducing mate selectivity and contributing to the higher intensity of social interactions when clerodanoids and their metabolites are involved (Paul and Müller 2022; Singh et al. 2024).

Contrary to our expectations, we found no detectable changes in CHC profiles following pharmacophagous uptake from leaves or contact with previously leaf-exposed conspecifics. In other insect species, such as the leaf beetle *Phaedon cochleariae* and many ant species, the CHC composition can be modified in response to the diet (Otte et al. 2018; Sprenger and Menzel 2020) and thus CHCs can show some plasticity. However, *A. rosae* do not feed on *A. reptans*, but only lick on the plant surface, which may not be sufficient to modify the composition of the CHC profile, at least within the tested timeframe. Moreover, it can be questioned whether the uptake of predominantly diterpenoids, but probably only few other metabolites or plant nutrients, can affect the CHC composition, unless under such circumstances energy is invested differently in metabolic pathways, i.e., clerodanoid and CHC metabolism, potentially leading to some trade-offs. The absence of effects on CHCs following conspecific contact in *A. rosae* may also be explained by the relatively short time between exposure and analysis in our experimental design. In natural populations of *A. rosae* with possibly frequent contact to *A. reptans*, long-term effects on the CHC profile might occur. In contrast, rapid CHC changes through direct transfer have been documented in Diptera species, such as *Drosophila serrata*, where significant compositional shifts can occur after sexual interaction within 15 min (Petfield et al. 2005). The lack of detectable CHC variation following conspecific contact in our laboratory population of *A. rosae* may also reflect the genetic homogeneity of our rearing stock, despite periodic gene pool renewal. Populations in nature likely exhibit greater genetic diversity that could manifest in a more pronounced CHC variation, as, for example, described in the ant species *Solenopsis geminata* (Hu et al. 2017). Future studies incorporating field-collected individuals from distinct populations of *A. rosae* may reveal differences in CHC profiles related to habitat and host plant-related traits. Understanding these characteristics in chemical communication networks and how they affect individual niches may also provide useful information for conservation and sustainable pest control.

## Supplementary Information

Below is the link to the electronic supplementary material.

Supplementary Material 1 (PDF 782 KB)

## Data Availability

Data are available via https://gitlab.ub.uni-bielefeld.de/lbrueggemann/semiochemical-perception-and-surface-compounds-in-the-turnip-sawfly.
