# Supplementary material for: The Roles of Semiochemical Perception and Surface Compounds in a Pharmacophagous Sawfly
Source: J Chem Ecol. 2025 Dec 15;51(6):122. doi: 10.1007/s10886-025-01676-1 (PMC12705808; doi:10.1007/s10886-025-01676-1)
Supplement: Supplementary file 1 — Supplementary Material 1 (PDF 782 KB) [file 10886_2025_1676_MOESM1_ESM.pdf]

SUPPLEMENTARY INFORMATION

For

The Roles of Semiochemical Perception and Surface Compounds in a Pharmacophagous Sawfly

Leon BRUEGGEMANN, Gina S. FLEER, Caroline MÜLLER

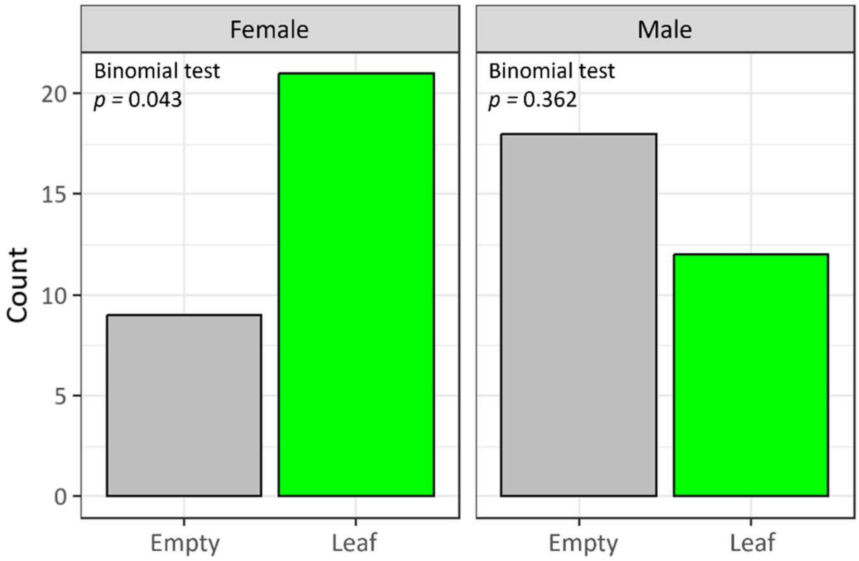

Fig. S1 Response of adult *Athalia rosae* to odors of cabbage leaves in the Y-tube olfactometer assay. Number of sawflies (n = 30 per sex) choosing either the empty side or the side with the cabbage leaf (binomial test).

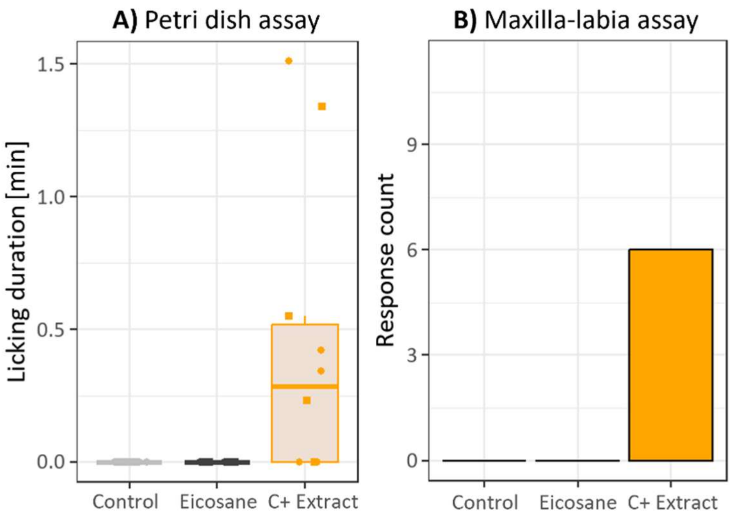

Fig. S2 Reaction of adult *Athalia rosae* to stimuli at closer distance in the Petri dish assay (A) and in the maxilla-labia response assay (B). A) Licking duration in Petri dish assay for female (dots) and male (square) adults (*n* = 10 in total). Boxplots show interquartile ranges (IQR, boxes) with medians, whiskers extent to ±IQR*1.5, and raw data points. B) Number of individuals showing maxilla-labia responses to different stimuli (out of 10 tested individuals). Control: dichloromethane, Eicosane: *n*-eicosane in dichloromethane (0.01 mg/ml), C+ Extract: surface wash from *A. rosae* that had prior contact to *Ajuga reptans* leaves.

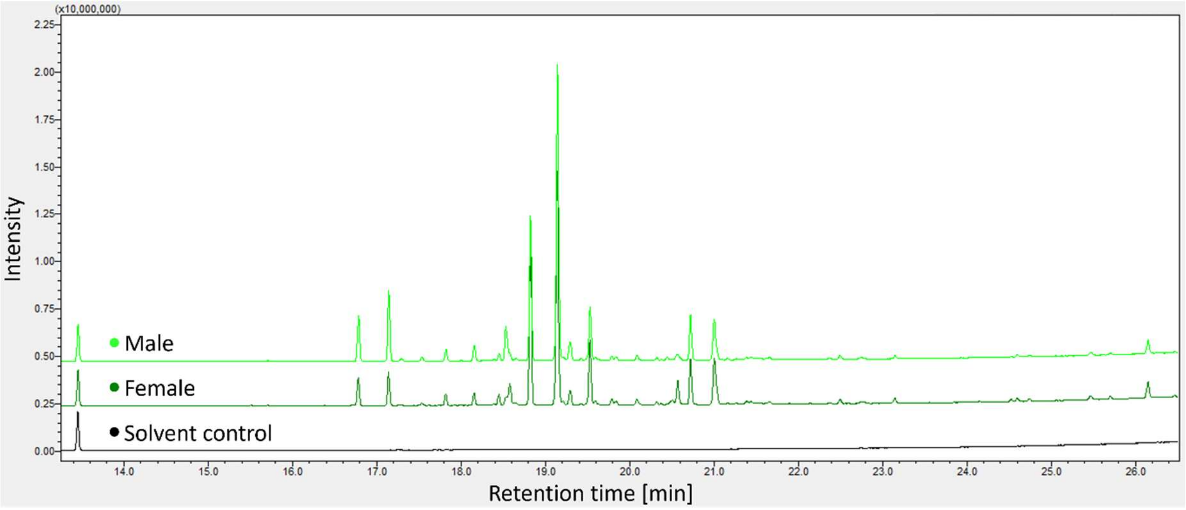

Fig. S3 Exemplary GC-MS chromatograms of surface washes of adult *Athalia rosae* that had not been exposed to *Ajuga reptans*. All samples contain *n*-eicosane as internal standard (at 13.5 min Rt).

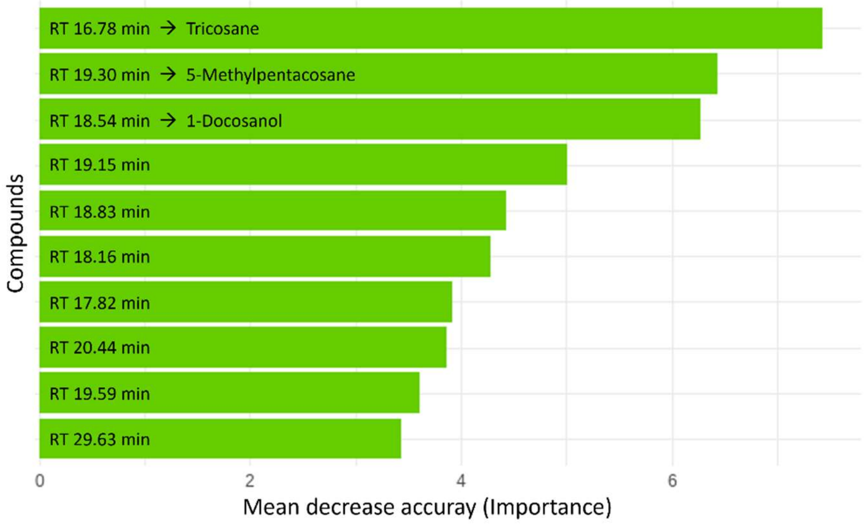

Fig. S4 Bar plot of random forest analysis of surface compounds of *Athalia rosae* measured via GC-MS, showing the 10 compounds with highest importance for sex separation. The three compounds with a mean decrease accuracy >5 were selected for further analysis. The peak areas were normalized to the internal standard and the body mass. The number of trees was set to 500.
